# A change in circulating chikungunya virus variant impacts *Aedes aegypti* vector competence and spatiotemporal distribution of disease in Malaysia

**DOI:** 10.1371/journal.pntd.0012632

**Published:** 2024-10-31

**Authors:** Izzati Kausar Azman, Yoke Fun Chan, Chong Long Chua, Zainal Abidin Abd Mutalib, Sarat Chandra Dass, Balvinder Singh Gill, Nor Hayati Ismail, Jenarun Jelip, Ming Keong Wan, Wenn-Chyau Lee, Indra Vythilingam, Luke Alphey, I-Ching Sam

**Affiliations:** 1 Department of Medical Microbiology, Faculty of Medicine, Universiti Malaya, Kuala Lumpur, Malaysia; 2 Population and Demographic Statistics Division, Department of Statistics, Putrajaya, Malaysia; 3 School of Mathematical & Computer Sciences, Heriot-Watt University Malaysia, Putrajaya, Malaysia; 4 Institute for Medical Research, National Institutes of Health, Shah Alam, Malaysia; 5 Molecular Unit, Public Health Laboratory Kota Bharu, Kota Bharu, Malaysia; 6 Vector-Borne Disease Section, Disease Control Division, Ministry of Health, Putrajaya, Malaysia; 7 Department of Parasitology, Universiti Malaya, Kuala Lumpur, Malaysia; 8 A*STAR Infectious Diseases Labs (A*STAR IDL), Agency for Science, Technology and Research (A*STAR), Singapore; 9 Department of Biology, University of York, York, United Kingdom; 10 York Biomedical Research Institute, University of York, York, United Kingdom; Texas A&M University, UNITED STATES OF AMERICA

## Abstract

**Background:**

In 2008–2010, Malaysia experienced a nationwide chikungunya virus (CHIKV) outbreak caused by the Indian Ocean lineage E1-226V (valine) variant, adapted to *Aedes albopictus*. In 2017–2022, transition to an E1-226A (alanine) variant occurred. *Ae*. *albopictus* prevails in rural areas, where most cases occurred during the E1-226V outbreak, while *Ae*. *aegypti* dominates urban areas. The shift in circulating CHIKV variants from E1-226V to E1-226A (2009–2022) was hypothesized to result in a transition from rural to urban CHIKV distribution, driven by differences in *Ae*. *aegypti* vector competence for the two variants. This study aimed to: (1) map the spatiotemporal spread of CHIKV cases in Malaysia between 2009–2022; and (2) compare replication of E1-226A and E1-226V variants in the midguts and head/thoraxes of *Ae*. *aegypti*.

**Methodology/Principal findings:**

Spatiotemporal analysis of national notified CHIKV case addresses was performed. Between 2009–2022, 12,446 CHIKV cases were reported, with peaks in 2009 and 2020, and a significant shift from predominantly rural cases in 2009–2011 (85.1% rural), to urban areas in 2017–2022 (86.1% urban; p<0.0001). Two *Ae*. *aegypti* strains, field-collected MC1 and laboratory Kuala Lumpur (KL) strains, were fed infectious blood containing constructed CHIKV clones, pCMV-p2020A (E1-226A) and pCMV-p2020V (E1-226V) to measure CHIKV replication by real-time PCR and/or virus titration. The pCMV-p2020A clone replicated better in *Ae*. *aegypti* cell line *Aag2* and showed higher replication, infection and dissemination efficiency in both *Ae*. *aegypti* strains, compared to pCMV-p2020V.

**Conclusions/Significance:**

This study revealed that a change in circulating CHIKV variants can be associated with changes in vector competence and outbreak epidemiology. Continued genomic surveillance of arboviruses is important.

## Author summary

Chikungunya virus (CHIKV) causes fever, rash and joint pain in humans. CHIKV is spread by bites of infected *Aedes* (*Ae*.) *aegypti* and *Ae*. *albopictus* mosquitoes. In 2008–2010, CHIKV with an E1-226V mutation became highly adapted to *Ae*. *albopictus* and caused global outbreaks. In Malaysia, these major outbreaks occurred mainly in rural areas where this mosquito is most common. We found that a resurgence of CHIKV in 2017–2022 was associated with a different variant with an E1-226A mutation, which had a greater ability to replicate in the *Ae*. *aegypti* mosquito than the previous E1-226V variant. This mosquito is found mainly in urban areas, and by looking at geographical locations of reported CHIKV cases, we found that the case distribution had also shifted from rural to predominantly urban areas in 2017–2022. Genetic surveillance of CHIKV and other mosquito-borne viruses is important to identify genetic changes that may affect virus ability to infect different vectors, and disease numbers and distribution.

## Introduction

Chikungunya virus (CHIKV) is an alphavirus which causes epidemics of fever, rash and prolonged joint pain, and is mainly transmitted by two mosquito vectors, *Aedes aegypti* and *Ae*. *albopictus*. Historically, CHIKV outbreaks have been primarily reported in urban locales, facilitated by the urban mosquito vector, *Ae*. *aegypti* [1]. In 2004, an Indian Ocean lineage (IOL) emerged from the East/Central and South African (ECSA) lineage of CHIKV; within this IOL, a CHIKV variant with an alanine to valine substitution in the E1 surface glycoprotein (E1-226V) emerged in La Reunion [2]. While the wild-type variant E1-226A appears better adapted to *Ae*. *aegypti* [3], the E1-226V variant showed increased adaptation to *Ae*. *albopictus*, playing a pivotal role in ensuing global epidemics of the IOL lineage over the following years, particularly in Asia [4].

The epidemic IOL E1-226V variant also showed greater adaptation to *Ae*. *albopictus* than the Asian lineage previously endemic in Malaysia [5]. Coupled with the widespread distribution of *Ae*. *albopictus* in rural areas, this led to an unprecedented nationwide outbreak of E1-226V variants in Malaysia between 2008–2010 which affected over 15,000 individuals mainly in rural regions and replaced the Asian lineage [6,7]. Our recent phylogenetic analysis of CHIKV variants from Malaysia and Asia between 2017 to 2021 revealed a shift back towards IOL variants with E1-226A from the previously predominant E1-226V variants. We hypothesised that this shift in virus variant resulted in a shift in the spatiotemporal distribution of CHIKV cases from rural to urban areas, where *Ae*. *aegypti* predominates.

To address this, we used geographical information system (GIS) analysis to map the distribution of CHIKV cases notified to the Ministry of Health, Malaysia (MOH) from 2009 to 2022. We then used mosquito infection to show that the current circulating CHIKV E1-226A variant has greater adaptation to *Ae*. *aegypti* than a corresponding E1-226V variant obtained by selective mutagenesis. The change in circulating variant to CHIKV E1-226A was associated with both an overall increase in reported CHIKV cases and a shift to an urban preponderance of the disease.

## Methodology

### Ethics statement

The study was approved by the Ministry of Health National Medical Research Register and Medical Research & Ethics Committee (protocol number NMRR ID-22-01234-GIY).

### Geospatial analysis of CHIKV cases

CHIKV became a notifiable disease in Malaysia from 2009 onwards under the administrative order of the Director-General of Health. Details of notified CHIKV cases from 2009 to 2022 were obtained from MOH, including residential addresses and date of onset of illness. Cases were defined by clinical symptoms and signs and epidemiological links to an outbreak area [8], and not all received laboratory confirmation. The geographic coordinates of the residential addresses were generated using the Geocode by Awesome Table plugin in Google Sheets using the World Geodetic System 1984 format, cross-checked with Google Maps, and visualised with QGIS v.3.28 [9] and ArcMap 10.7.1 (Earth Science Resource Institute, USA). The geocoded addresses were categorized into urban and rural settings by the Department of Statistics, Malaysia, based on established criteria such as population density and economic activities [10]. National and state population numbers were taken from the 2010 and 2020 censuses [10,11]. SaTScan v.10.1.2 (https://www.satscan.org/) was used to identify significant CHIKV clusters by space-time scan analysis [12]. The optimum maximum spatial cluster size was determined through Gini coefficient calculation in R Statistical Software v.4.3.1 [13] and set at 5% of the total population size.

### Phylogenetic analysis

A total of 153 complete CHIKV genome sequences excluding vaccine and cloning vector strains, available as of March 2023, were retrieved from GenBank. These included eight recent IOL isolates detected in Malaysia from 2017 to 2021 and previously sequenced in our laboratory (S1 Table). Sequences of 11,218 nucleotides were trimmed and aligned using Geneious Prime 2023.1.2 (Biomatters, New Zealand). The general time-reversible substitution model with gamma distribution and invariant sites (GTR + G + I) was chosen using ModelFinder [14]. A phylogenetic tree was constructed with BEAST v2.7.5 [15] using an optimised relaxed molecular clock and Bayesian skyline coalescent tree prior, and run with 100 million chain length to achieve effective sample sizes >200 for all parameters. The resulting tree was visualized using FigTree version 1.4.4 (http://tree.bio.ed.ac.uk/software/figtree/).

### Mosquito collection

Vector competence of two *Ae*. *aegypti* strains was examined. An *Ae*. *aegypti* laboratory colony (Kuala Lumpur [KL] strain) was sourced from the Institute of Medical Research, originating from Kuala Lumpur in the 1970s. Field *Ae*. *aegypti* mosquitoes (MC1 strain) were collected in 2021 and 2022 from Petaling Jaya in Selangor state. Kuala Lumpur and Petaling Jaya are part of the greater Klang Valley metropolitan area. The MC1 field strain was collected from one of the 39 sites around Malaysia where *Wolbachia*-infected (wAlbB strain) *Ae*. *aegypti* has been released as a biocontrol measure to curb transmission of dengue virus [16,17]. Oviposition traps using hay infusion to attract mosquitoes for egg-laying were placed in moist and dark areas both outdoors and indoors. Collected eggs were transported to the insectarium and reared through the stages of larvae, pupae and eventually adult mosquitoes within a week. *Ae*. *aegypti* was identified based on morphology [18]. Female mosquitoes were provided with a blood meal and separated into individual containers secured with a net. Eggs were hatched and colonised under controlled conditions at 28 ± 1°C, 80 ± 10% humidity and 12 hours: 12 hours light-dark cycle. Using PCR [19], we confirmed the presence of *w*AlbB in the MC1 line and its absence in the KL line before infection work.

### Cell culture

Baby hamster kidney (BHK-21; ATCC no. CCL-10) and *Ae*. *aegypti Aag*2 cell lines (a kind gift from the Roslin Institute, University of Edinburgh, UK) were utilized for *in vitro* infection. BHK-21 cells were cultured in Glasgow’s minimal essential medium (Gibco, USA) supplemented with 5% fetal bovine serum (FBS; Bovogen, Australia), 100 U/ml penicillin and 100 μg/ml streptomycin (Gibco), 2 mM L-Glutamine (Gibco), 20 mM 4-(2-hydroxyethyl)-1-piperazineethanesulfonic acid (Gibco) and 10% tryptose phosphate broth (TPB; Sigma, USA). The cells were incubated at 37°C in a humidified incubator with 5% CO_2_. *Aag*2 cells were grown in Leibovitz’s L-15 medium (Sigma) supplemented with 10% FBS, 100 U/ml penicillin and 100 μg/ml streptomycin and 1% TPB, and incubated at 28°C.

### Construction of CHIKV infectious clones

The CHIKV strain MY/2020/3092435 (IOL lineage, GenBank accession MW557661) was originally isolated from a human CHIKV case in Kuala Lumpur in 2020 and carries E1-226A. The entire genome was amplified and substituted into the infectious clone ICRES1 with a CMV-promoter-driven vector system (IOL lineage, based on KT449801, isolated in La Reunion in 2006) [20]. The CHIKV whole genome was divided into five fragments and amplified with specific primers (S2 Table). Each fragment was then inserted into a vector using CloneJET PCR Cloning Kit (Thermo Scientific, USA). The CHIKV fragments (S1 Fig) were inserted into different vectors based on their restriction enzyme (RE) sites, with blunt end fragments 1, 4 and 5 inserted into the pJET cloning vector, while sticky end fragments 2 and 3 were inserted into a pSK vector (generously provided by Andres Merits, University of Tartu). To introduce a point mutation leading to an amino acid change from A to V at position 226 of the E1 glycoprotein, site-directed mutagenesis using conventional PCR was employed on fragment 5 using the forward primer 5’-(GCACGTGTACCGTACCC)-3’ and reverse primer 5’-(ACAGCCGGTCTCTGCAGT)-3’.

The pCMV-ICRES1 plasmid was used as a backbone for inserting the plasmids containing fragments 1 to 5. Synthesized stuffer sequences, designed to include recognition sites for RE, were ligated with the pCMV-ICRES1 backbone, facilitating the subsequent insertion of fragments. For optimal plasmid yield required for downstream work, the ligation mixtures of pCMV-p2020A (E1-226A) and pCMV-p2020V (E1-226V) were transformed into XL-10 Gold (Agilent, Australia).

Both infectious clones were introduced into BHK-21 cells using the Gene Pulser Xcell electroporation system (Bio-Rad, USA). Following an incubation period of 48 hours, P0 rescued viruses were harvested. The virus titer was determined using the tissue culture infectious dose 50 assay (TCID_50_). To generate P1 virus stock, P0 rescued viruses were used to infect BHK-21 cells at a multiplicity of infection (MOI) of 0.1.

### CHIKV replication in *Aag2* cells

The replication kinetics of pCMV-p2020A and pCMV-p2020V viruses were compared in *Aag2* cells. Cell monolayers were exposed to the viruses at MOI of 0.1. Virus supernatant was collected from individual wells at 0, 24, 48, 72 and 96 hours post-infection (hpi) and the virus titers were determined using the TCID_50_ assay. Two biological replicates were performed for each of two independent experimental series.

### CHIKV replication kinetics in *Ae*. *aegypti*

Infection work was conducted in an arthropod containment level 2 facility. The KL (laboratory strain) and MC1 (ninth generation) strain of *Ae*. *aegypti* mosquitoes, aged 3 to 5 days, were fed via a Hemotek feeding system (Discovery Workshop, UK) with blood from volunteers. Engorged mosquitoes were kept in the same controlled conditions, and unfed mosquitoes were discarded. The appropriate oral infectious doses (OID) for E1-226A and E1-226V viruses were determined, considering their potentially different vector competence. Virus stocks were diluted and mixed with blood at a ratio of 1:10 into final concentrations of 2 log_10_ TCID_50_/ml, 4 log_10_ TCID_50_/ml and 6 log_10_ TCID_50_/ml. For each oral infectious dose, 20 whole mosquitoes were collected and homogenized at 0 and 7 days post-infection (dpi). Virus isolation and conventional PCR using CHIKV-F (5’-(CAGCAAGAAAGGCAAGTGTGC)-3’) and CHIKV-R (5’-(TGACTATGTGGTCCTTCGGAGG)-3’) primers [5] were employed to detect CHIKV in whole mosquitoes. The infection rate was defined as the proportion of whole mosquitoes harbouring PCR-detectable or culturable virus (at 7 dpi) divided by the total number of tested engorged mosquitoes successfully fed a blood meal. Virus culture has the advantage of confirming infectious virus, while PCR has greater sensitivity.

Probit analysis was used to assess differences between the OID for each virus variant. For subsequent experiments, both groups of mosquitoes were fed with OIDs determined for each virus to ensure >60% infection of mosquitoes. At each timepoint of 0, 3, 5, 7, 10 and 14 dpi, 20 female mosquitoes were harvested. The midgut and the combined head and thorax (head/thorax) were dissected to measure virus titers using TCID_50_ assays and identify the presence of virus using qPCR. The midgut infection rate was defined as the number of mosquitoes in which CHIKV was detectable in the midguts divided by the total number of tested engorged mosquitoes successfully fed a blood meal. The dissemination efficiency was calculated as the number of mosquitoes with detectable CHIKV in heads/thoraxes divided by the total number of engorged mosquitoes tested [21]. Dissecting needles were immersed in 70% ethanol and then in 10% bleach between mosquitoes to eliminate potential carry-over of RNA and virus. Negative control mosquitoes received a clean blood meal devoid of any viruses. Twenty mosquitoes from the negative control group were collected at each timepoint for dissection. Organs were individually homogenized in 1.5 ml zirconium beads tubes (Benchmark Scientific, USA), pre-filled with 500 μl of MEM supplemented and 2% amphotericin B. Viral RNA was extracted using the IndiSpin Pathogen Kit (QIAGEN, Germany). The extracted samples and homogenates were stored in -80°C for further analysis. A summary of the recommended data standard for vector competence experiments [22] is shown in S3 Table.

### Real-time PCR assay of E1 gene

Real-time PCR assay was utilized to determine the presence of viral RNA. *In vitro* transcribed RNA controls were synthesised using the clinical isolate MY/08/065 (GenBank accession number FN295485) to generate a standard curve for the E1 gene.

RNA was serially diluted in 10-fold increments ranging from 10−10^9^ copies/μL using nuclease-free water. The RT-qPCR reaction comprised 4.0 μl of 5X RT-qPCR reaction mix (Roche, Switzerland), 400 μM of CHIKV E1-F (5’-(AAGCTYCGCGTCCTTTACCAAG)-3’) and CHIKV E1-R (5’-(CCAAATTGTCCYGGTCTTCCT)-3’) primers, 250 μM E1-probe (5’-(CCAATGTCYTCMGCCTGGACACCTTT)-3’) [23], 1 μl of RNA template, 0.1 μl of 200X RT-Enzyme solution and nuclease-free water into a final reaction volume of 15 μl. A no template control was included. Amplification was performed using the StepOne Plus real-time PCR system (Applied Biosystems, USA), with reverse transcription at 50°C for 10 minutes, then denaturation at 95°C for 30 seconds. This was followed by 40 cycles of denaturation at 95°C for 5 seconds and annealing/extension at 60°C for 30 seconds. The detection limit of the assay was 100 copies of RNA per reaction, corresponding to a cycle threshold value of 36. The CHIKV E1 expression in all samples was determined.

### Statistical analysis

Statistical analyses were performed and graphs were constructed using GraphPad Prism version 7.00 for Windows (GraphPad Software, USA). CHIKV infection and dissemination efficiency rates were compared using Fisher’s exact test. Differences in virus titers between mosquitoes were analysed with the Mann Whitney U test. Differences were considered statistically significant if the two-tailed P-value < 0.05.

## Results

### Evolutionary dynamics and emergence of CHIKV IOL variants in Malaysia

**Fig 1:**
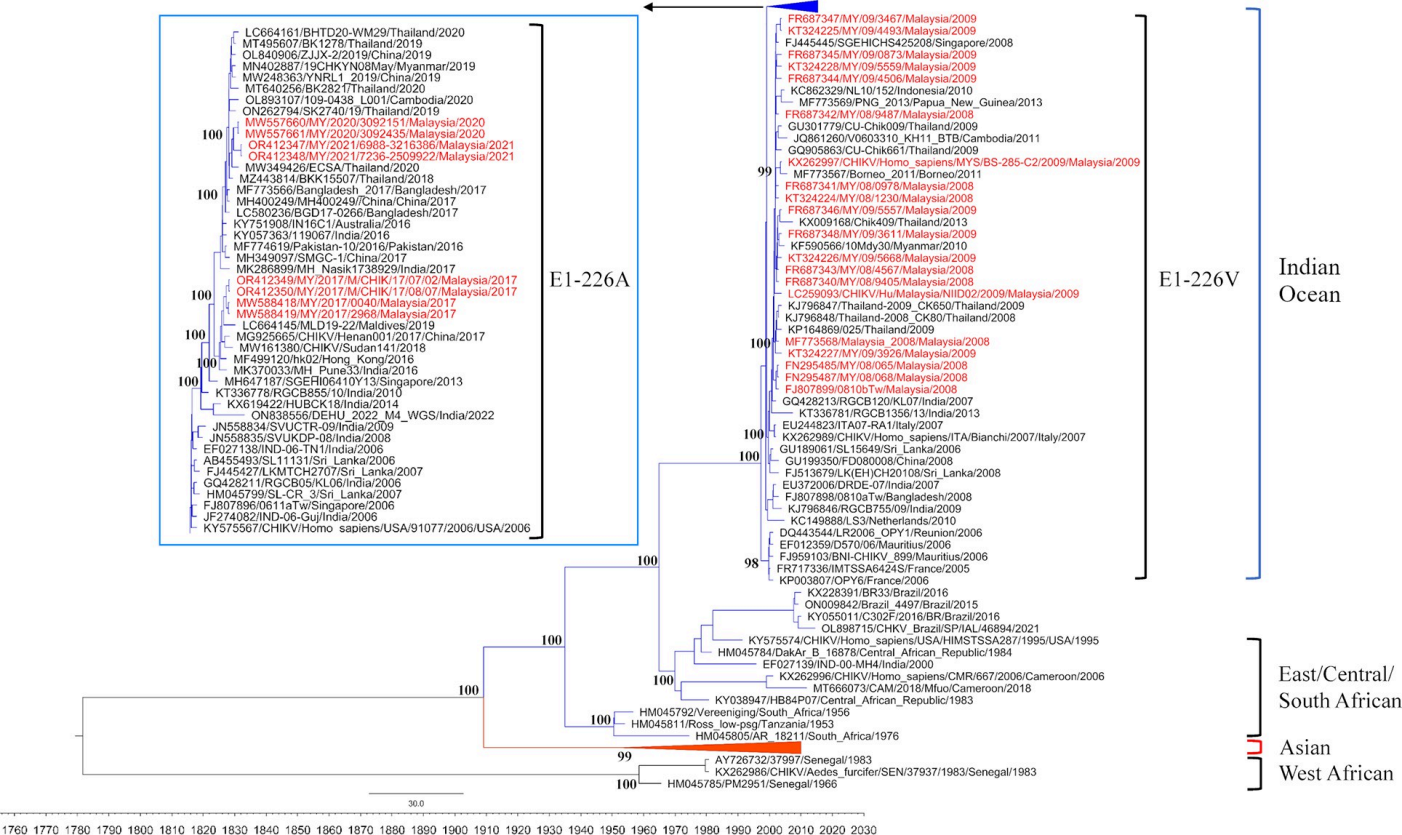
Phylogenetic tree of 153 CHIKV whole genome sequences. Phylogenetic analysis reveals a shift in CHIKV IOL variants in Malaysia from E1-226V (2008–2009) to E1-226A (2017–2021). The tree was constructed using the general time-reversible model with gamma distribution and invariant sites (GTR + G + I). Sequences are represented in the format: accession number/strain name/country of origin/year of sample. The estimated posterior probability values are shown at key nodes. Malaysian sequences are shown in red.

The phylogenetic tree revealed four distinct lineages of CHIKV: IOL, ECSA, Asian and West African (Fig 1). The IOL with E1-226A and E1-226V variants emerged in 2006 from Reunion. The predominant variants during subsequent global spread carried E1-226V, including outbreaks in Malaysia in 2008–2009 and the Asian region. A notable shift in CHIKV dynamics occurred in Asia around 2013–2014, when IOL E1-226A variants were increasingly detected, and clearly became predominant in Asia by 2016. These include eight Malaysian sequences obtained between 2017 and 2021, which are found in two separate clusters (one in 2017, and one in 2020–2021) containing other sequences from Asia, with robust posterior probability. These Malaysian E1-226A variants also clustered separately from the 2008–2009 Malaysian E1-226V sequences.

### Spatiotemporal shift of CHIKV from rural to urban areas

**Fig 2:**
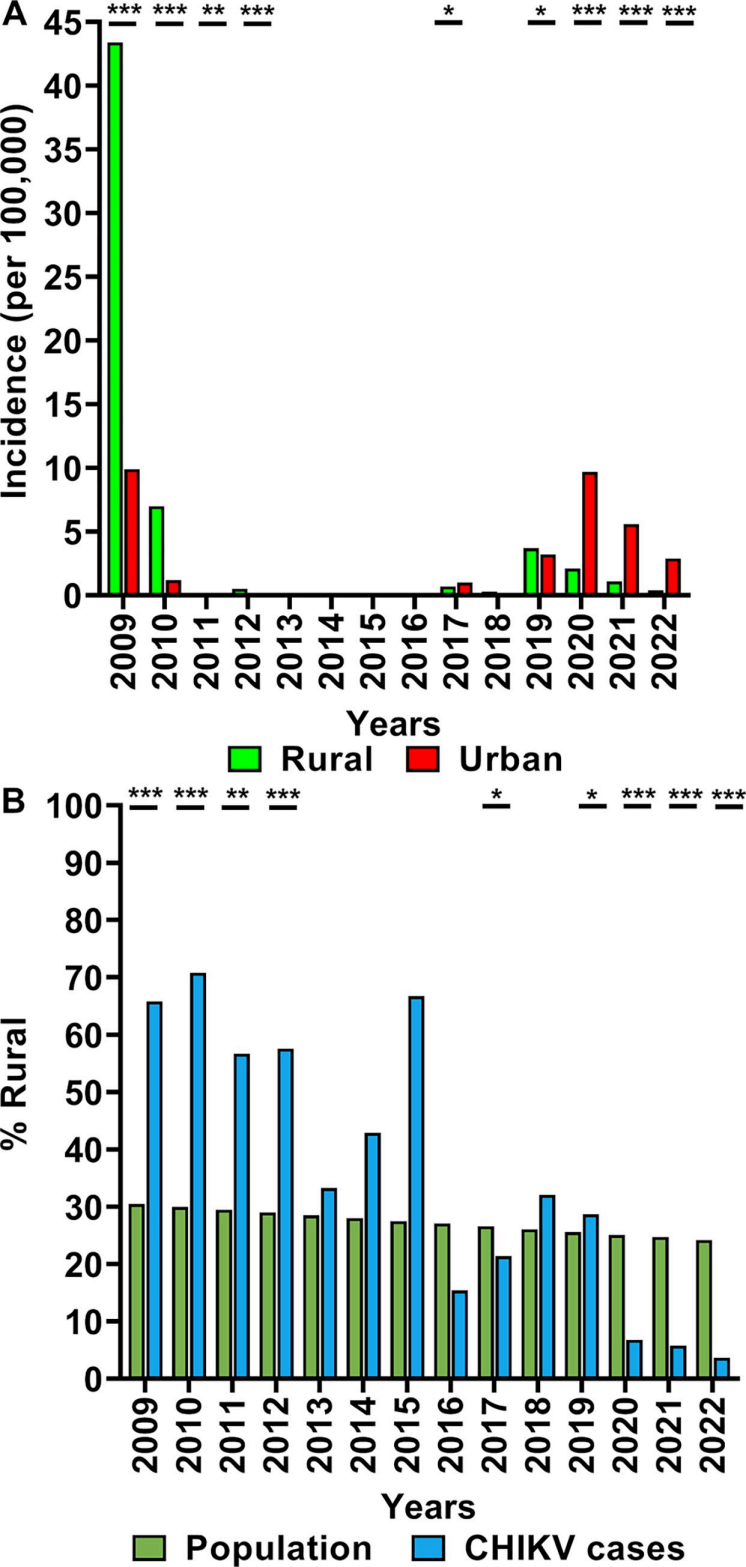
Urban/rural incidences of CHIKV cases and overall urban/rural populations of Malaysia from 2009 to 2022. (A) Urban and rural incidences per 100,000 population of CHIKV cases; (B) rural proportions of CHIKV cases and the overall Malaysian population. Statistical differences were assessed using chi-square tests; p-values shown are p<0.05 (*), p<0.01 (**) and p<0.001(***).

In total, 12,442 CHIKV cases were notified to MOH between 2009–2022 (S2 Fig). The rural/urban incidence rates of CHIKV cases for Malaysia are shown in Fig 2A. Malaysia experienced a significant CHIKV nationwide outbreak in 2008–2010. However, data from 2008 is unavailable as CHIKV became formally notifiable only from 2009. The peak annual national incidence was 20.1 per 100,000 in 2009 (43.4 per 100,000 rural and 9.9 per 100,000 urban population). There was a drastic decline of reported CHIKV incidence to <0.4 per 100,000 between 2011 and 2016. Slight increases occurred in 2017 and 2018, before more substantial outbreaks were observed from 2019, including the second-highest number of annual cases during the study period in 2020 (2,503 cases). CHIKV incidence then rose sharply to a range of 0.3–7.8 per 100,000 per year between 2017–2022.

Spatiotemporal mapping of cases indicated a shift from mainly rural areas all around Malaysia in 2009–2010, particularly in East Malaysia, to mainly urban areas in the western states of Peninsular Malaysia in recent years (S3 Fig). This was confirmed by higher rural incidence of cases in 2009–2010 shifting to mainly urban incidence in recent years 2017–2022. A possible confounder is the gradually increasing urbanisation of Malaysia between 2009–2022, with overall rural populations decreasing from 30.5% to 24.2% (Fig 2B). Nevertheless, over the same period, the rural proportion of CHIKV cases decreased much more drastically from 65.8% to 3.7% (p<0.0001 for trend).

**Fig 3:**
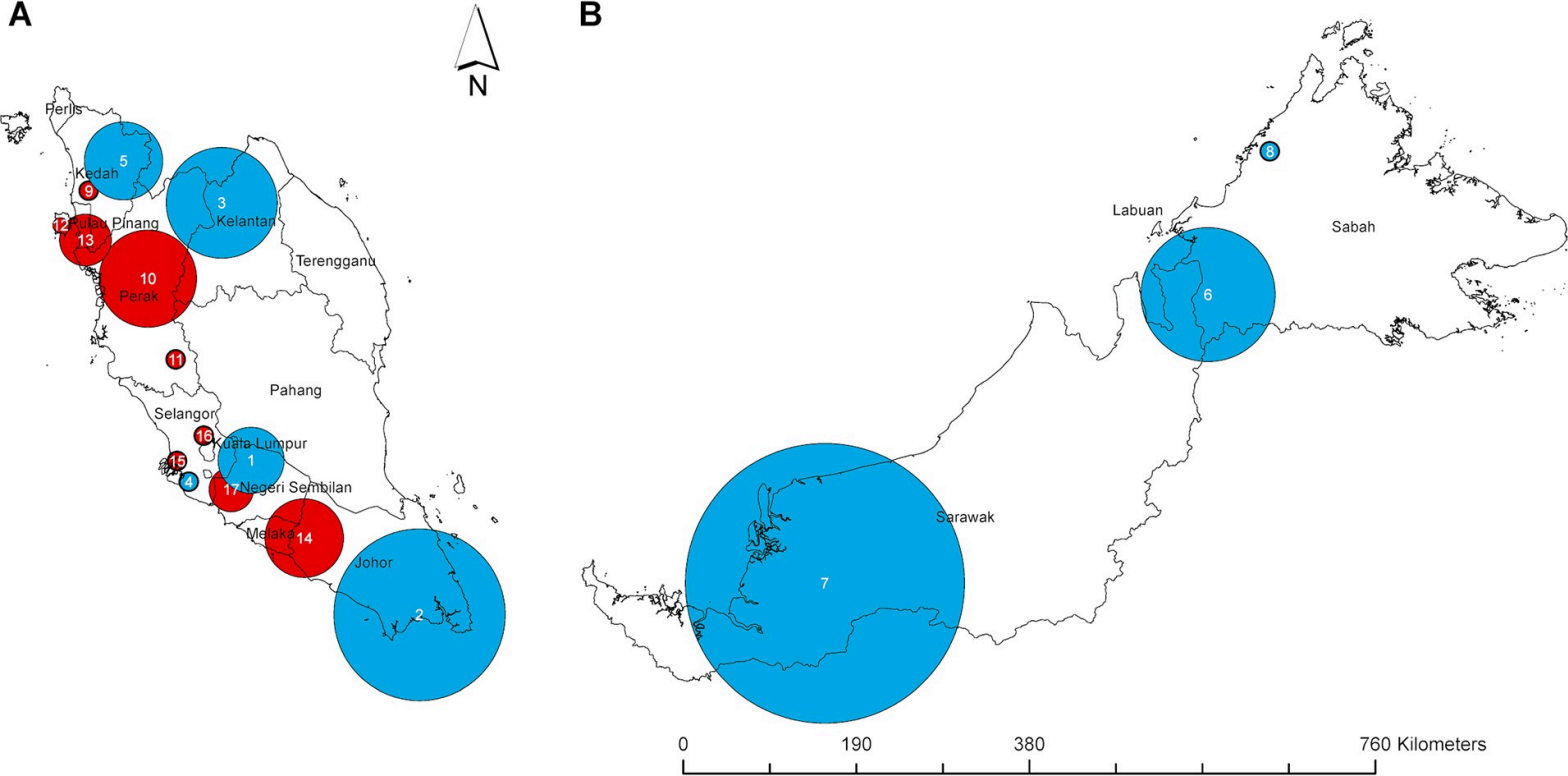
Spatiotemporal cluster maps of CHIKV cases, encompassing 17 clusters across Malaysia, 2009 to 2022. (A) Peninsular (West) Malaysia and (B) East Malaysia. Data was generated using SaTScan with the maximum spatial cluster size set at 5% of population size. Seventeen distinctive clusters in nine states were identified, each denoted with a circle representing the best-encompassing radius. Blue clusters occurred from 2008–2010 (E1-226V variant circulating); red clusters occurred from 2017–2022 (E1-226A variant circulating). The basemap of Malaysia was obtained from the Database of Global Administrative Areas (https://gadm.org/download_country36.html) and is freely available for academic use.

Space-time scan analysis identified 17 spatiotemporal clusters of CHIKV cases between 2009 and 2022 (Fig 3 and Table 1), comprising 6,643 (53%) of the total 12,442 cases. The initial clusters were identified in December 2008 in Negeri Sembilan, Johor and Kelantan (clusters 1–3) across distinct regions of Peninsular Malaysia. In early 2009, there were clusters in Selangor (cluster 4) and a large cluster of 625 cases in Kedah (cluster 5). The first cluster in East Malaysia was detected in Sabah in June 2010 (cluster 6), followed by the largest CHIKV cluster of 1,947 cases in Sarawak (cluster 7). Of the nine clusters occurring between 2008–2010, seven involved a higher proportion of rural cases, ranging from 69.6–96.9%.

**Table 1:** Spatiotemporal clustering of 6,643 CHIKV cases in 17 clusters in Malaysia, 2009 to 2022.

Cluster (states, no. of districts involved)	Cluster period	Center (radius) of cluster	No. of cases	% of all clustered cases	Log likelihood ratio	Rural:urban cases (%)
Cluster 1 (Negeri Sembilan, 3)	29/12/2008 to 29/3/2009	3.04°N, 102.11°E (36.1 km)	56	0.8	70	45:55
Cluster 2 (Johor, 7)	29/12/2008 to 12/4/2009	1.52°N, 103.78°E (93.9 km)	94	1.4	145	80:20
Cluster 3 (Kelantan, 6)	29/12/2008 to 23/8/2009	5.58°N, 101.82°E (60.6 km)	468	7	1,139	86:14
Cluster 4 (Selangor, 1)	19/1/2009 to 15/3/2009	2.83°N, 101.50°E (0 km)	159	2.4	581	97:3
Cluster 5 (Kedah, 5)	20/4/2009 to 17/5/2009	5.99°N, 100.85°E (2.7 km)	625	9.4	3,398	97:3
Cluster 6 (Sabah, 4)	3/8/2009 to 22/11/2009	4.67°N, 115.72°E (73.3 km)	97	1.5	284	87:13
Cluster 7 (Sarawak, 19)	14/9/2009 to 31/1/2010	1.82°N, 111.94°E (153.1 km)	1,947	29.3	7,525	70:30
Cluster 8 (Sabah, 1)	14/6/2010 to 4/7/2010	6.08°N, 116.33°E (0 km)	117	1.8	631	92:7
Cluster 9 (Kedah, 1)	6/3/2017 to 28/5/2017	5.70°N, 100.51°E (0 km)	97	1.5	217	2:98
Cluster 10 (Perak, 4)	10/6/2019 to 20/9/2020	4.83°N, 101.09°E (53.2 km)	1,210	18.2	2,602	3:97
Cluster 11 (Perak, 1)	9/9/2019 to 19/1/2020	4.03°N, 101.37°E (0 km)	298	4.5	1,139	40:60
Cluster 12 (Pulau Pinang, 2)	11/5/2020 to 30/8/2020	5.36°N, 100.23°E (8.3 km)	898	13.5	3,364	7:93
Cluster 13 (Pulau Pinang, 2; Kedah, 2; Perak, 1)	15/6/2020 to 17/1/2021	5.21°N, 100.48°E (28.3 km)	119	1.8	101	3:97
Cluster 14 (Johor, 2; Melaka, 2; Negeri Sembilan, 1)	28/9/2020 to 27/6/2021	2.27°N, 102.64°E (43.1 km)	160	2.4	127	16:84
Cluster 15 (Selangor, 1)	25/1/2021 to 14/2/2021	3.03°N, 101.38°E (0 km)	43	0.6	96	0:100
Cluster 16 (Selangor, 1)	9/8/2021 to 26/12/2021	3.28°N, 101.65°E (0 km)	175	2.6	301	1:99
Cluster 17 (Negeri Sembilan, 2)	20/12/2021 to 6/3/2022	2.75°N, 101.92°E (24.3 km)	56	0.8	70	0:100

Data was generated using SaTScan with the maximum spatial cluster size set at 5% of population size. The radius value corresponds to the inter-centroid separation between two or more districts, and a radius of 0 km signifies the presence of a solitary district within the cluster. All clusters shown are statistically significant (p< 0.0001).

There were no clusters observed between 2011 to 2016. Nine clusters were detected between 2017 and 2022, all affecting predominantly urban populations in Peninsular (West) Malaysia. There was a single small cluster in Kedah (cluster 9) between March to May 2017, with all subsequent clusters occurring from 2019. Clusters 10–13 in Perak, Pulau Pinang and Kedah were all located in the northern region of Peninsular Malaysia. CHIKV clusters spread southwards from September 2020, and were detected in Johor, Melaka, Selangor and Negeri Sembilan (clusters 14–17).

In summary, the phylogenetic and GIS analyses indicate that the shift of CHIKV cases from mainly rural areas around the country (particularly in East Malaysia) to mainly urban areas in the western states of Peninsular Malaysia was temporally associated with a change in the predominant circulating CHIKV variant from E1-226V to E1-226A.

### The E1-226A variant has faster replication than the E1-226V variant in *Ae*. *aegypti* (*Aag2*) cells

**Fig 4:**
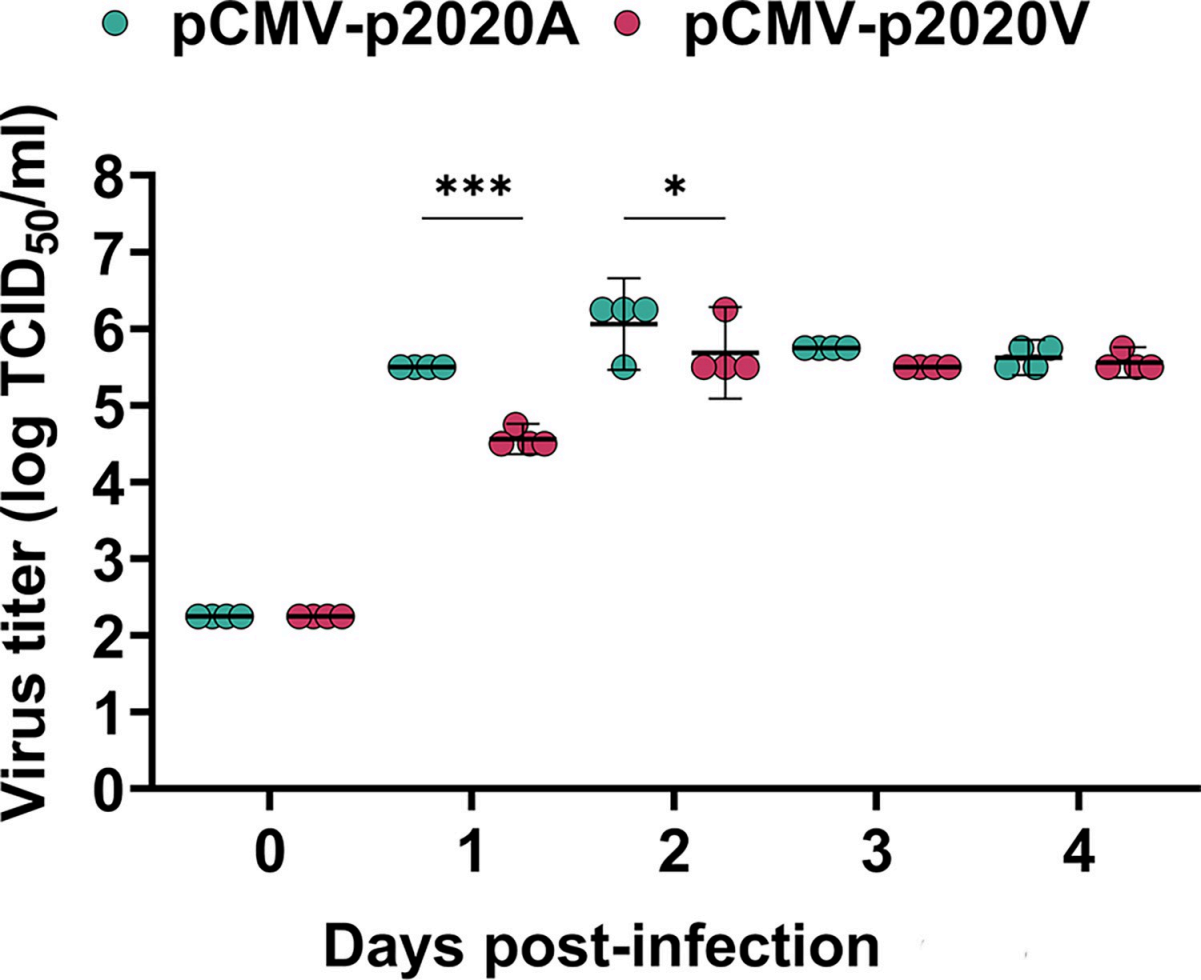
Replication kinetics of pCMV-p2020A and pCMV-p2020V in *Aag2* cell line. Virus titers of pCMV-p2020A (E1-226A) and pCMV-p2020V (E1-226V) are shown up to 4 dpi. Statistical comparisons were performed using two-way ANOVA with the Bonferroni post-hoc test. Bars represent means and 95% confidence intervals. The p-values shown are: p<0.05 (*) and p<0.001(***).

We next determined the fitness of the two variants in *Ae*. *aegypti* cell lines and mosquitoes. The replication kinetics of pCMV-p2020A (E1-226A) and pCMV-p2020V (E1-226V) were first evaluated in *Aag2* cells (Fig 4). pCMV-p2020A displayed more rapid growth within the first 24 hpi, and both reached their maximum viral titers at 48 hpi (5.7–6.1 log TCID_50_/ml) and plateaued up to 96 hpi. There were significantly higher titers (p<0.05) of pCMV-p2020A than pCMV-p2020V at 24 and 48 hpi.

### Lower oral infectious doses required by E1-226A variants to infect *Ae*. *aegypti*

**Fig 5:**
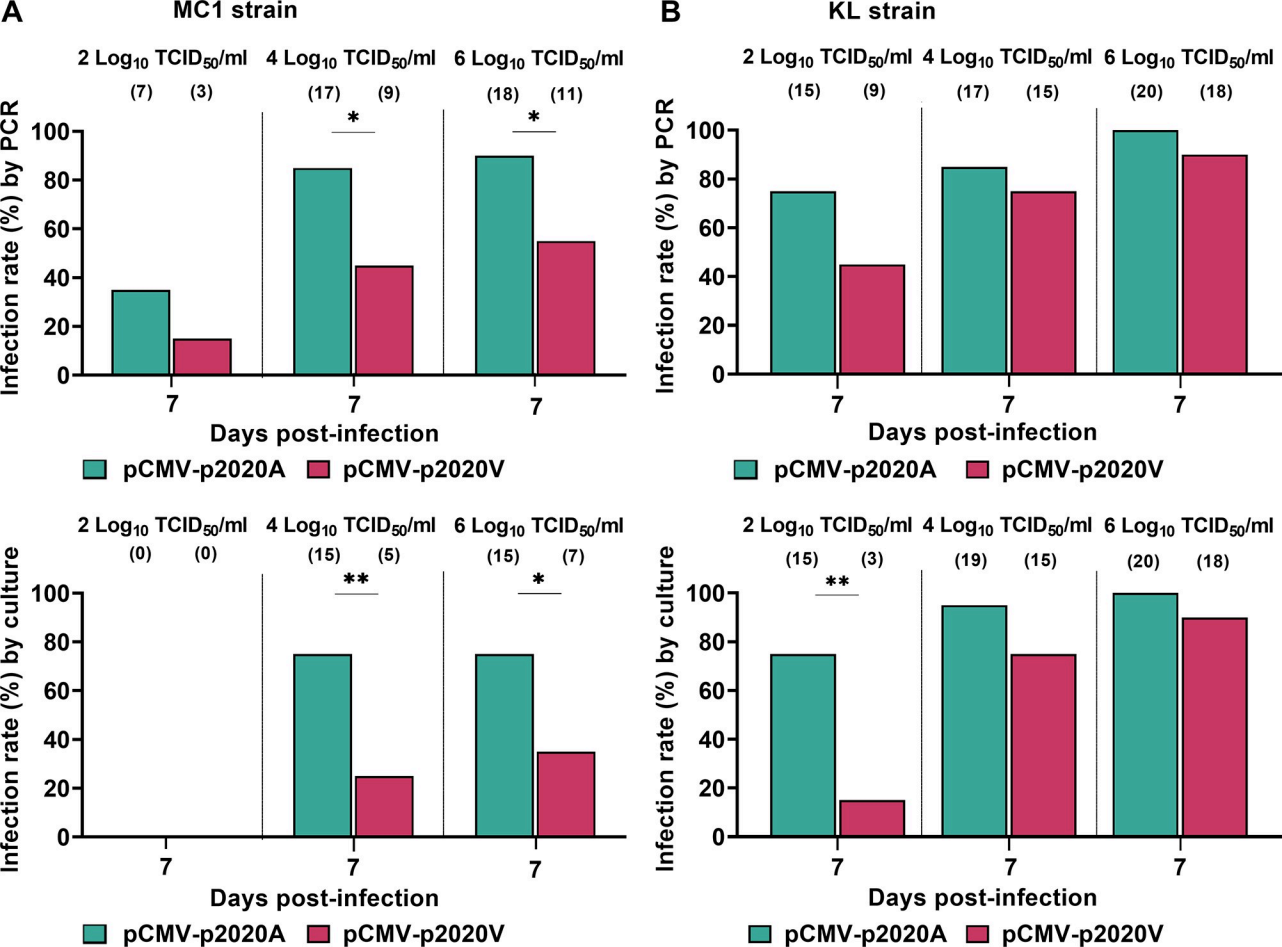
Infection rates of pCMV-p2020A and pCMV-p2020V in *Ae*. *aegypti* at 7 dpi, using different oral infectious doses. pCMV-p2020A and pCMV-p2020V were orally administered to MC1 mosquitoes at 2, 4, and 6 log TCID_50_/ml, and the infection rates were assessed by PCR detection and virus isolation at 7 dpi (A). Infection rates for KL mosquitoes were also determined using PCR detection and virus isolation (B). Virus detections were also carried out at day 0 and confirmed the presence of virus/infectious virus in ingested blood meals. Infection rates were compared with Fisher’s exact test. Numbers of infected mosquitoes are shown in brackets, out of a total of 20 mosquitoes tested at each timepoint. The p-values shown are: p<0.05 (*) and p<0.01 (**).

Both strains of *Ae*. *aegypti* mosquitoes (MC1 and KL) were orally infected with either pCMV-p2020A or pCMV-p2020V at three different doses (2, 4, and 6 log TCID_50_/ml) to determine the optimal dose for infecting mosquitoes for subsequent experiments and to compare OID (Fig 5). Whole mosquitoes were collected and homogenized at 7 dpi to determine infection rates by PCR and virus isolation.

In MC1 mosquitoes fed infectious doses of 4 log TCID_50_/ml and 6 log TCID_50_/ml, pCMV-p2020A exhibited significantly higher infection rates at 7 dpi (Fig 5A). In KL mosquitoes, pCMV-p2020A showed higher infection rates at 7 dpi at all three infectious doses, but the only statistically significant difference was between the rates measured by virus isolation at the 2 log TCID_50_/ml dose (Fig 5B). At the lowest infectious dose of 2 log TCID_50_/ml, there was detectable replication of both variants in KL mosquitoes at 7 dpi (Fig 5B), but not in MC1 mosquitoes (Fig 5A).

Probit analysis was used to determine the minimum oral viral dose required to infect a specific proportion of the mosquito population. The OIDs were consistently lower for pCMV-p2020A than pCMV-p2020V in both mosquitoes (Table 2), and this achieved statistical significance for OID_50_ and OID_75_ in MC1 mosquitoes, and OID_75_ for KL mosquitoes. For further infection experiments, in MC1 mosquitoes, we selected doses of 4 log TCID_50_/ml for pCMV-p2020A and 6 log TCID_50_/ml for pCMV-p2020V. In KL mosquitoes, a dose of 2 log TCID_50_/ml was utilized for pCMV-p2020A, and 4 log TCID_50_/ml for pCMV-p2020V. These OIDs correlated to OID_64_ to OID_73_, and ensured high infection rates. Overall, the lower OIDs of pCMV-p2020A showed that the E1-226A variant had greater ability to orally infect *Ae*. *aegypti* compared to the E1-226V variant, with the effect being more pronounced in the MC1 field strain.

**Table 2:** Oral infectious doses of CHIKV to infect *Ae*. *aegypti* mosquitoes.

Oral infectious dose	OID, log_10_ TCID_50_/ml (95% confidence intervals)			
	MC1 mosquitoes		KL mosquitoes	
	pCMV-p2020A	pCMV-p2020V	pCMV-p2020A	pCMV-p2020V
50	2.3* (0.6, 3.3)	5.0* (4.0, 6.3)	0.4 (-3.1, 1.8)	2.4 (0.7, 3.3)
75	4.2* (3.1, 5.5)	6.9* (5.7, 9.3)	2.3* (0.4, 3.5)	4.1* (3.2, 5.4)
90	5.9 (4.7, 8.1)	8.6 (7.0, 12.2)	4.0 (2.7, 5.8)	5.6 (4.6, 8.1)
99	8.8 (7.0, 13.1)	11.5 (9.1, 17.4)	6.9 (5.2, 11.3)	8.3 (6.5, 13.2)

Differences between pCMV-p2020A and pCMV-p2020V at a particular OID were compared; p<0.05 (*).

### E1-226A variants show greater infectivity, replication and dissemination in both MC1 and KL mosquitoes

We next determined the replication of the CHIKV variants in midguts (demonstrating infection) and heads/thoraxes (demonstrating dissemination) of MC1 and KL mosquitoes up to 14 dpi.

**Fig 6:**
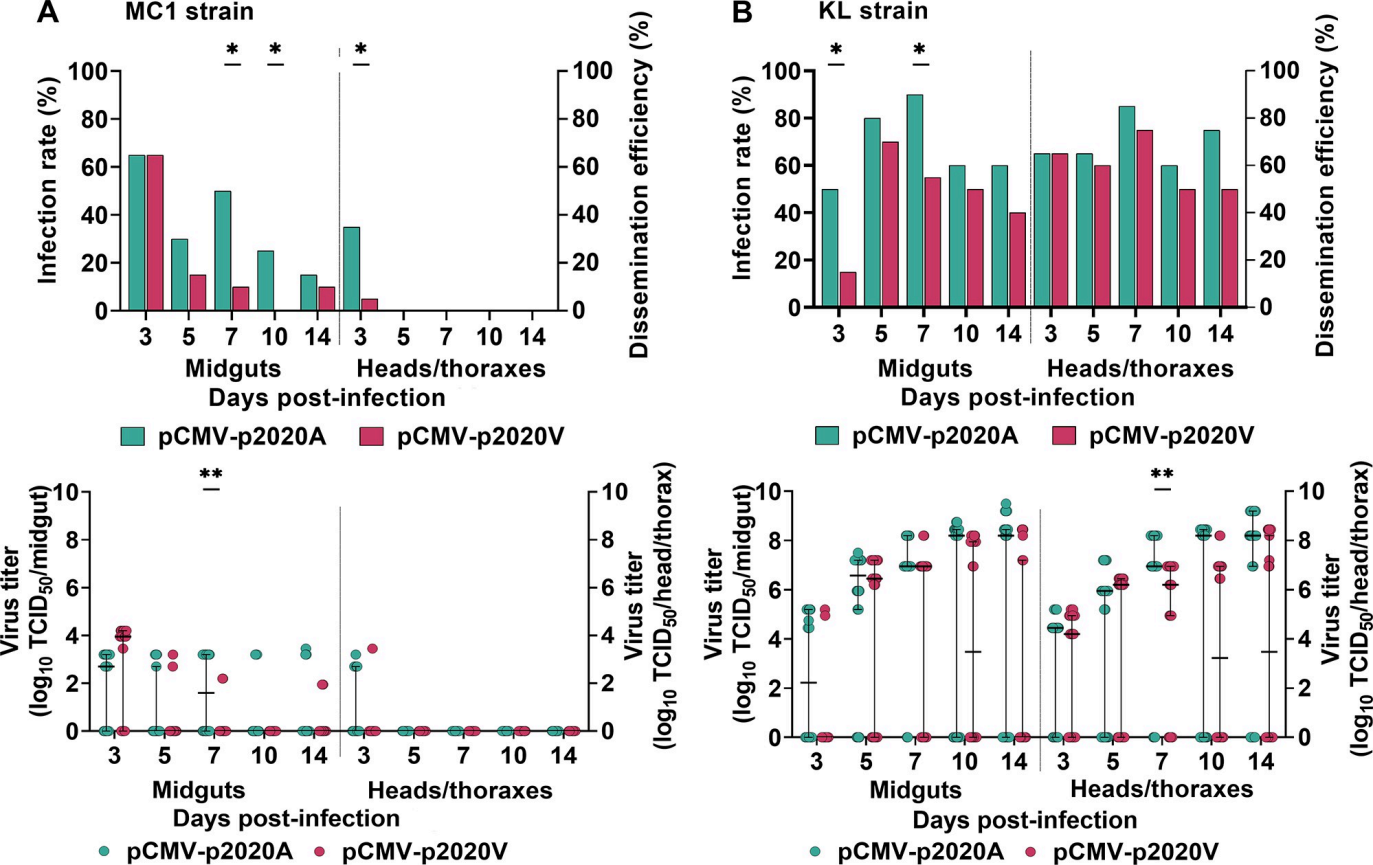
The replication kinetics of pCMV-p2020A and pCMV-p2020V in midguts and heads/thoraxes of *Ae*. *aegypti*. Infection rate in midguts (determined by virus titration) and dissemination efficiency in heads/thoraxes (virus titers), measured in MC1 mosquitoes (A) and KL mosquitoes (B). Virus detections were also carried out at day 0 and confirmed the presence of virus/infectious virus in ingested blood meals. Rates were compared with Fisher’s exact test and median virus titers assessed using Mann Whitney U test. Twenty mosquitoes were tested at each time-point. The p-values shown are: p<0.05 (*) and p<0.01 (**).

In MC1 mosquitoes, despite the lower oral infecting dose, pCMV-p2020A showed higher infection rates (7 and 10 dpi) and higher virus titers (7 dpi) in midguts (Fig 6A). In heads/thoraxes at 3 dpi, the dissemination efficiency of pCMV-p2020A was significantly higher at 35% compared to 5% for pCMV-p2020V. Out of 20 head/thorax samples from MC1 mosquitoes with pCMV-p2020A, seven samples (35%) exhibited virus titers ranging from 2.5 to 3.5 log TCID_50_ per thorax/head, whereas only one sample (5%) showed detectable virus for pCMV-p2020V, although these differences did not reach statistical significance. These observations indicate that pCMV-p2020A demonstrates better replication fitness than pCMV-p2020V in MC1 mosquitoes. No further dissemination to heads/thoraxes were observed for both viruses from 5–14 dpi.

In KL mosquitoes, infection rates were significantly higher for pCMV-p2020A compared to pCMV-p2020V at 3 and 7 dpi in midguts (Fig 6B). Both viruses showed increasing midgut and head/thorax titers up to 14 dpi, reaching maximum titers of around 8 log TCID_50_/ml (Fig 6B). Despite starting off with a 2 log lower oral infectious dose, pCMV-p2020A reached similar levels to pCMV-p2020V, and even achieved significantly higher virus titers in 7 dpi in heads/thoraxes, demonstrating better ability of pCMV-p2020A to establish and sustain infection in KL mosquitoes. Taken together, these findings indicate that the presence of E1-226A in CHIKV enhances viral replication or adaptation in both strains of *Ae*. *aegypti*.

## Discussion

The first laboratory-confirmed outbreaks of CHIKV in Malaysia occurred in 1999 and 2006, and were caused by the Asian lineage endemic in Asia [24,25]. A large nationwide outbreak occurred between 2008–2010, predominantly in rural areas, caused by epidemic IOL E1-226V viruses [7]. After several years of very low incidence, a CHIKV resurgence started in 2017, with the reemergence of the E1-226A variant associated with a shift in cases from rural to urban areas. We showed that the pCMV-p2020A (E1-226A) variant exhibited significantly higher infection, replication and dissemination in *Ae*. *aegypti* than pCMV-p2020V (E1-226V).

Global travel contributes to CHIKV emergence, as travellers from endemic areas introduce the virus to new regions, with CHIKV showing the ability to adapt to local *Aedes* vectors [26]. The initial epidemic IOL lineage in 2004 carried E1-226A [2], before the E1-226V mutation arose by convergent evolution [27]. E1-226V variants show greater adaptation to *Ae*. *albopictus* [4], aided by second step mutations in E2 (such as E2-K252Q and E2-L210Q) which confer further adaptation [28]. This has driven extensive epidemics of IOL viruses in rural and suburban areas of Asia since 2005. An epistatic interaction between E1-226 and E1-98T residues likely constrained endemic Asian CHIKV viruses from acquiring E1-226V-associated adaptation to *Ae*. *albopictus*, allowing IOL E1-226V variants to predominate within this niche [3]. However, in 2010, E1-226A viruses with specific adaptive mutations E1-K211E and E2-V264A that enhance virus fitness and transmission in *Ae*. *aegypti* were first detected in India. These viruses caused resurgent outbreaks in India (2016), Pakistan (2016), Bangladesh (2017), Thailand (2018) and Myanmar (2019) [29]. These E1-226A, E1-K211E and E2-V264A mutations have also been identified in all Malaysian sequences reported since 2017. Thus, the recent spike in Malaysian CHIKV E1-226A cases has mirrored the situations in other Asian countries. Meanwhile, different lineages dominate outside Asia; for example, the Americas have experienced outbreaks of the Asian-American (until 2018) and ECSA-American lineages, the latter being distinct from the IOL and becoming dominant in recent years [30].

The mechanisms by which the E1-226 residue impacts vector competence are not clearly understood. During fusion with mammalian cells, CHIKV envelope proteins intricately interact with specific host cell membrane lipid components, particularly cholesterol and sphingomyelin [31–33]. Specific amino acid variations, including E1-226V, enhance fusion interaction with cholesterol-rich membranes [32]. pH levels influence the fusion process, with optimal efficiency typically below 5.6 [31]. E1-226V mutants exhibit increased sensitivity to pH and require a higher cholesterol level for fusion compared to the wild-type CHIKV with E1-226A in Sf21 insect cells [34] and C6/36 cells [35], although this does not directly correlate with phenotype of *Ae*. *albopictus* adaptation [35]. Arboviral infection of mosquitoes involves overcoming two key tissue barriers–the midgut and salivary glands. In *Ae*. *albopictus*, selection of the E1-226V variant over E1-226A appears to occur during midgut infection [36]. More research on the mechanisms driving E1-226A enhanced adaptability to *Ae*. *aegypti* is required.

Our study showed that the Malaysian outbreaks in 2008–2010 mainly occurred in rural areas. These rural outbreaks were particularly reported in regions with palm oil and rubber plantations [37]. *Ae*. *aegypti* are highly domesticated vectors which favour artificial containers and indoor settings, while *Ae*. *albopictus* prefers natural sites with vegetation. Numerous studies in Malaysia have found *Ae*. *albopictus* to be prevalent in rural areas, while both *Ae*. *aegypti* (predominantly) and *Ae*. *albopictus* are found in urban and suburban regions [38–41]. This aligns with the findings that rural outbreaks from 2008 to 2010 were mainly driven by CHIKV E1-226V adapted to *Ae*. *albopictus* [42], while recent urban cases are likely linked to CHIKV E1-226A adapted to *Ae*. *aegypti*. Recent outbreaks in Thailand in 2018–2019 were also caused by E1-226A variant harbouring E1-K211E and E2-V264A, and largely occurred in urban areas with *Ae*. *aegypti* [43]. In West Bengal, India, two variants of the IOL of CHIKV circulated concurrently. The E1-226A variant was identified in Kolkata, a city with a high prevalence of *Ae*. *aegypti*, in 2011 and 2012 [44]. Conversely, the CHIKV E1-226V variant was found in rural areas in West Bengal between 2006 and 2012 [45]. Thus, the epidemiological distribution of CHIKV cases can be affected by the predominant *Aedes* vector (which can vary within an area) and the vector-specific adaptation of the causative outbreak viral strain.

Apart from the emergence of highly *Ae*. *aegypti*-adapted E1-226A variants, another important possible reason for the apparent shift of CHIKV to urban areas in this study could be the pre-existing immunity in rural areas resulting from previous outbreaks, which may confer lifelong protection [46]. Although the peak reported CHIKV incidence in 2009 was only 20.1 per 100,000, under-reporting is likely, as a seroprevalence study conducted during the first outbreak year in 2008 reported CHIKV seropositivity rates of up to 19.8% in rural areas in Negeri Sembilan state [37]. In Brazil [47] and Thailand [48] which have experienced multiple outbreaks, newer CHIKV cases were more likely to occur in areas less affected by previous outbreaks, suggesting spatial heterogeneity of CHIKV spread and variations in population immunity.

There was inter-strain variability in *Ae*. *aegypti*, with the MC1 field strain showing more pronounced differences in competence for pCMV-p2020A, while the KL strain, a well-established laboratory colony, appears highly susceptible to both CHIKV variants. As mentioned earlier, the MC1 field strain is *Wolbachia*-infected (*w*AlbB strain), and was collected from one of the release sites in the *Wolbachia* national program [16,17], while the KL strain is uninfected. *Wolbachia* is a maternally inherited endosymbiotic bacterium which inhibits replication of arboviruses like dengue virus, Zika virus, CHIKV and yellow fever virus in mosquitoes [49–51]. Infection with *Wolbachia* strain *w*Mel can inhibit CHIKV in *Ae*. *aegypti* [51] and reduce CHIKV incidence in the field in Brazil [52]. However, there is limited research showing *w*AlbB efficacy against CHIKV, including an *in vitro* study in C6/36 (*Ae*. *albopictus*) cells [53] and recent *in vivo* work from Singapore, where *w*AlbB-infected male *Ae*. *aegypti* have been released to suppress mosquito populations [54]. Apart from differences in laboratory adaptation and genetic background, the presence of *w*AlbB may contribute to inter-strain differences in vector competence; notably, in our study, CHIKV was not detected in heads and thoraxes of MC1 mosquitoes beyond 3 dpi. Considering that virus inhibition by *Wolbachia* is dependent on both virus and mosquito genetic backgrounds [54] and that pathogen enhancement has been occasionally reported [55], it is important future work to formally assess the effectiveness of *w*AlbB-infected *Ae*. *aegypti* from the field against different circulating CHIKV strains in Malaysia.

Several limitations must be acknowledged in this study. First, there is a scarcity of sequenced Malaysian CHIKV variants, which may be due to undersampling, undetected circulation, or a true low incidence. Nevertheless, the dominant Malaysian variants in 2008–2010 and then in 2017–2022 correlated with those reported in Asia during the same periods. There are likely other factors, notably previous population immunity, which influenced cases during these two time periods, which were beyond the remit of this study. For example, dengue incidence notably declined sharply during the COVID-19 pandemic [56], yet Southeast Asia saw increases in CHIKV infections, despite both viruses sharing the same mosquito vectors. Saliva was not tested during mosquito infection work, although it is the best indicator of transmission. Future vector competence experiments should include saliva testing and the use of a wider range of clinically relevant OIDs for finer differentiation of transmissibility risk.

In conclusion, the change in circulating CHIKV from the E1-226V to the E1-226A variant in 2017 led to increased *Ae*. *aegypti* vector competence, and coincided with the transition of CHIKV cases from rural to urban settings where this vector predominates. The currently prevalent CHIKV variant in Malaysia, E1-226A, coupled with ongoing urbanization trends, raises concerns for potential large urban outbreaks in the future. Ongoing arbovirus genomic surveillance is crucial, given the dynamic nature of viral genetic changes that may influence vector adaptation, thereby potentially impacting the incidence, location of diseases and effectiveness of control measures.

## Supporting information

S1 Fig Construction of infectious clones pCMV-p2020A (E1-226A) and pCMV-p2020V (E1-226V). (PDF)

S2 Fig Incidence of notified CHIKV cases in Malaysia, 2009–2022. (PDF)

S3 Fig Spatiotemporal dynamics of notified CHIKV cases in Malaysia between 2009–2022. (PDF)

S1 Table Sequences from recently reported CHIKV cases in Malaysia. (PDF)

S2 Table PCR primers for amplification of five CHIKV fragments for cloning. (PDF)

S3 Table Minimum data standard for vector competence experiments. (XLSX)

S1 Data Experimental data for Figs 2, 4, 5 and 6, and S2 Fig. (XLSX)

## References

[1] Volk S. M., Chen R., Tsetsarkin K. A., Adams A. P., Garcia T. I., Sall A. A., et al. Genome-scale phylogenetic analyses of chikungunya virus reveal independent emergences of recent epidemics and various evolutionary rates. J Virol. 2010; 84(13): 6497–6504. doi: 10.1128/JVI.01603-09 . 20410280 PMC2903258

[2] Schuffenecker I, Iteman I, Michault A, Murri S, Frangeul L, Vaney MC, et al. Genome microevolution of chikungunya viruses causing the Indian Ocean outbreak. PLoS Med. 2006; 3(7): e263. doi: 10.1371/journal.pmed.0030263 . 16700631 PMC1463904

[3] Tsetsarkin KA, Chen R, Sherman MB, Weaver SC. Chikungunya virus: evolution and genetic determinants of emergence. Curr Opin Virol. 2011; 1(4): 310–317. doi: 10.1016/j.coviro.2011.07.004 . 21966353 PMC3182774

[4] Vazeille M, Moutailler S, Coudrier D, Rousseaux C, Khun H, Huerre M, et al. Two chikungunya isolates from the outbreak of La Reunion (Indian Ocean) exhibit different patterns of infection in the mosquito, *Aedes albopictus*. PLoS One. 2007; 2(11): e1168. doi: 10.1371/journal.pone.0001168 . 18000540 PMC2064959

[5] Sam IC, Loong SK, Michael JC, Chua CL, Wan Sulaiman WY, Vythilingam I, et al. Genotypic and phenotypic characterization of chikungunya virus of different genotypes from Malaysia. PLoS One. 2012; 7(11): e50476. doi: 10.1371/journal.pone.0050476 . 23209750 PMC3507689

[6] Dass S, Ngui R, Gill BS, Chan YF, Wan Sulaiman WY, Lim YAL, et al. Spatiotemporal spread of chikungunya virus in Sarawak, Malaysia. Trans R Soc Trop Med Hyg. 2021; 115(8): 922–931. doi: 10.1093/trstmh/trab053 . 33783526

[7] Sam IC, Chan YF, Chan SY, Loong SK, Chin HK, Hooi PS, et al. Chikungunya virus of Asian and Central/East African genotypes in Malaysia. J Clin Virol. 2009; 46(2): 180–183. doi: 10.1016/j.jcv.2009.07.016 . 19683467

[8] Ministry of Health, Malaysia. Guidelines for the prevention and control of chikungunya disease in Malaysia. Putrajaya, Malaysia: Ministry of Health, 2012. Available from: http://www.moh.gov.my/moh/resources/ auto%20download%20images/589d71fcbb60f.pdf.

[9] GIS.org. QGIS, Geographical Information System, Open Source Geospatial Foundation Project. 2023. Available from: http://www.qgis.org.

[10] Department of Statistics, Malaysia. Population and housing census of Malaysia—population and basic demographic characteristics 2010. Putrajaya, Malaysia: Department of Statistics. 2011.

[11] Department of Statistics, Malaysia. Population and housing census of Malaysia—population and basic demographic characteristics 2020. Putrajaya, Malaysia: Department of Statistics. 2023.

[12] Kulldorff M, Heffernan R, Hartman J, Assunção R, Mostashari F. A space-time permutation scan statistic for disease outbreak detection. PLoS Med. 2005; 2(3): e59. doi: 10.1371/journal.pmed.0020059 . 15719066 PMC548793

[13] R Core Team. R: A language and environment for statistical computing. 2023. R Foundation for Statistical Computing, Vienna, Austria. Available from: https://www.R-project.org/.

[14] Kalyaanamoorthy S, Minh BQ, Wong TKF, von Haeseler A, Jermiin LS. ModelFinder: fast model selection for accurate phylogenetic estimates. Nat Methods. 2017; 14(6): 587–589. doi: 10.1038/nmeth.4285 . 28481363 PMC5453245

[15] Bouckaert R, Vaughan TG, Barido-Sottani J, Duchêne S, Fourment M, Gavryushkina et al. BEAST 2.5: An advanced software platform for Bayesian evolutionary analysis. PLoS Comput Biol. 2019; 15(4): e1006650. doi: 10.1371/journal.pcbi.1006650 . 30958812 PMC6472827

[16] Hoffmann AA, Ahmad NW, Wan MK, Cheong YL, Ahmad NA, Golding N, et al. Introduction of *Aedes aegypti* mosquitoes carrying *w*AlbB *Wolbachia* sharply decreases dengue incidence in disease hotspots. iScience. 2024; 27(2): 108942. doi: 10.1016/j.isci.2024.108942 . 38327789 PMC10847733

[17] Ahmad NA, Mancini MV, Ant TH, Martinez J, Kamarul GMR, Nazni WA, et al. *Wolbachia* strain *w*AlbB maintains high density and dengue inhibition following introduction into a field population of *Aedes aegypti*. Philos Trans R Soc Lond B Biol Sci. 2021; 376(1818): 20190809. doi: 10.1098/rstb.2019.0809 . 33357050 PMC7776933

[18] Saleh F, Kitau J, Konradsen F, Alifrangis M, Lin CH, Juma S, et al. Habitat characteristics for immature stages of *Aedes aegypti* in Zanzibar city, Tanzania. J Am Mosq Control Assoc. 2018; 34(3): 190–200. doi: 10.2987/17-6709.1 . 31442169

[19] Zhou W, Rousset F, O’Neil S. Phylogeny and PCR-based classification of *Wolbachia* strains using *wsp* gene sequences. Proc Biol Sci. 1998; 265(1395): 509–515. doi: 10.1098/rspb.1998.0324 . 9569669 PMC1688917

[20] Pohjala L, Utt A, Varjak M, Lulla A, Merits A, Ahola T, et al. Inhibitors of alphavirus entry and replication identified with a stable chikungunya replicon cell line and virus-based assays. PLoS One. 2011; 6(12): e28923. doi: 10.1371/journal.pone.0028923 . 22205980 PMC3242765

[21] Vega-Rúa A, Zouache K, Girod R, Failloux AB, Lourenço-de-Oliveira R. High level of vector competence of *Aedes aegypti* and *Aedes albopictus* from ten American countries as a crucial factor in the spread of chikungunya virus. J Virol. 2014; 88(11): 6294–6306. doi: 10.1128/JVI.00370-14 . 24672026 PMC4093877

[22] Wu VY, Chen B, Christofferson R, Ebel G, Fagre AC, Gallichotte EN, et al. A minimum data standard for vector competence experiments. Sci Data 2022; 9(1): 634. doi: 10.1038/s41597-022-01741-4 . 36261651 PMC9582208

[23] Pastorino B, Bessaud M, Grandadam M, Murri S, Tolou HJ, Peyrefitte CN. Development of a TaqMan RT-PCR assay without RNA extraction step for the detection and quantification of African chikungunya viruses. J Virol Methods. 2005; 124(1–2): 65–71. doi: 10.1016/j.jviromet.2004.11.002 . 15664052

[24] Lam SK, Chua KB, Hooi PS, Rahimah MA, Kumari S, Tharmaratnam M, et al. Chikungunya infection—An emerging disease in Malaysia. Southeast Asian J Trop Med Public Health. 2001; 32(3): 447–451. . 11944696

[25] AbuBakar S, Sam IC, Wong PF, MatRahim N, Hooi PS, Roslan N. Reemergence of endemic chikungunya, Malaysia. Emerg Infect Dis. 2007; 13(1): 147–149. doi: 10.3201/eid1301.060617 . 17370532 PMC2725805

[26] Matusali G, Colavita F, Bordi L, Lalle E, Ippolito G, Lippi G, Castilletti C. Tropism of the chikungunya virus. Viruses. 2019; 11(2): 175. doi: 10.3390/v11020175 . 30791607 PMC6410217

[27] de Lamballerie X, Leroy E, Charrel RN, Ttsetsarkin K, Higgs S, Gould EA. Chikungunya virus adapts to tiger mosquito via evolutionary convergence: a sign of things to come? Virol J. 2008; 5: 33. doi: 10.1186/1743-422X-5-33 . 18304328 PMC2266737

[28] Tsetsarkin KA, Chen R, Yun R, Rossi SL, Plante KS, Guerbois M, et al. Multi-peaked adaptive landscape for chikungunya virus evolution predicts continued fitness optimization in *Aedes albopictus* mosquitoes. Nat Commun. 2014; 5: 4084. doi: 10.1038/ncomms5084 . 24933611 PMC7091890

[29] Khongwichit S, Chansaenroj J, Chirathaworn C, Poovorawan Y. Chikungunya virus infection: molecular biology, clinical characteristics, and epidemiology in Asian countries. J Biomed Sci. 2021; 28(1): 84. doi: 10.1186/s12929-021-00778-8 . 34857000 PMC8638460

[30] de Souza WM, Ribeiro GS, de Lima STS, de Jesus R, Moreira FRR, Whittaker C, et al. Chikungunya: a decade of burden in the Americas. Lancet Reg Health Am. 2024; 30: 100673. doi: 10.1016/j.lana.2023.100673 . 38283942 PMC10820659

[31] van Duijl-Richter MK, Hoornweg TE, Rodenhuis-Zybert IA, Smit JM. Early events in chikungunya virus infection-from virus cell binding to membrane fusion. Viruses. 2015; 7(7): 3647–3674. doi: 10.3390/v7072792 . 26198242 PMC4517121

[32] Hoornweg TE, Mareike KSVDR, Nuñez NVA, Albulescu IC, Hemert MJV, Smit JM. Dynamics of chikungunya virus cell entry unraveled by single-virus tracking in living cells. J Virol. 2016; 90(9): 4745–4756. doi: 10.1128/JVI.03184-15 . 26912616 PMC4836339

[33] Blijleven JS, Bouma EM, van Duijl-Richter MKS, Smit JM, van Oijen AM. Cooperative chikungunya virus membrane fusion and its substoichiometric inhibition by CHK-152 antibody. Viruses. 2022; 14(2): 270. doi: 10.3390/v14020270 . 35215863 PMC8877538

[34] Kuo SC, Chen YJ, Wang YM, Tsui PY, Kuo MD, Wu TY, et al. Cell-based analysis of chikungunya virus E1 protein in membrane fusion. J Biomed Sci. 2012; 19(1): 44. doi: 10.1186/1423-0127-19-44 . 22520648 PMC3384457

[35] Tsetsarkin KA, McGee CE, Higgs S. Chikungunya virus adaptation to *Aedes albopictus* mosquitoes does not correlate with acquisition of cholesterol dependence or decreased pH threshold for fusion reaction. Virol J. 2011; 8: 376. doi: 10.1186/1743-422X-8-376 . 21801412 PMC3162544

[36] Arias-Goeta C, Mousson L, Rougeon F, Failloux AB. Dissemination and transmission of the E1-226V variant of chikungunya virus in *Aedes albopictus* are controlled at the midgut barrier level. PLoS One. 2013; 8(2): e57548. doi: 10.1371/journal.pone.0057548 . 23437397 PMC3578806

[37] Azami NA, Salleh SA, Shah SA, Neoh HM, Othman Z, Zakaria SZ, et al. Emergence of chikungunya seropositivity in healthy Malaysian adults residing in outbreak-free locations: chikungunya seroprevalence results from the Malaysian cohort. BMC Infect Dis. 2013; 13: 67. doi: 10.1186/1471-2334-13-67 . 23379541 PMC3651385

[38] Chang MS, Jute N. Distribution and density of *Aedes aegypti* (L) and *Aedes albopictus* (Skuse) in Sarawak. Med J Malaysia. 1982; 37(3): 205–210. . 7176997

[39] Chen CD, Benjamin S, Saranum MM, Chiang YF, Lee HL, Nazni WA, et al. Dengue vector surveillance in urban residential and settlement areas in Selangor, Malaysia. Trop Biomed. 2005; 22(1): 39–43. . 16880752

[40] Saifur RG, Hassan AA, Dieng H, Salmah MR, Saad AR, Satho T. Temporal and spatial distribution of dengue vector mosquitoes and their habitat patterns in Penang Island, Malaysia. J Am Mosq Control Assoc. 2013; 29(1): 33–43. doi: 10.2987/12-6228R.1 . 23687853

[41] Ab Hamid N, Mohd Noor SN, Isa NR, Md Rodzay R, Bachtiar Effendi AM, Hafisool AA, et al. Vertical infestation profile of *Aedes* in selected urban high-rise residences in Malaysia. Trop Med Infect Dis. 2020; 5(3): 114. doi: 10.3390/tropicalmed5030114 . 32646026 PMC7557596

[42] Rozilawati H, Faudzi AY, Rahidah AA, Azlina AH, Abdullah AG, Amal NM, et al. Entomological study of chikungunya infections in the state of Kelantan, Malaysia. Indian J Med Res. 2011; 133(6): 670–673. . 21727669 PMC3135998

[43] Khongwichit S, Chansaenroj J, Thongmee T, Benjamanukul S, Wanlapakorn N, Chirathaworn C, et al. Large-scale outbreak of chikungunya virus infection in Thailand, 2018–2019. PLoS One. 2021; 16(3): e0247314. doi: 10.1371/journal.pone.0247314 . 33690657 PMC7946318

[44] Pramanik MK, Aditya G, Raut SK. Seasonal prevalence of *Aedes aegypti* immatures in Kolkata, India. Southeast Asian J Trop Med Public Health. 2007; 38(3): 442–447. . 17877217

[45] Taraphdar D, Chatterjee S. Molecular characterization of chikungunya virus circulating in urban and rural areas of West Bengal, India after its re-emergence in 2006. Trans R Soc Trop Med Hyg. 2015; 109(3): 197–202. doi: 10.1093/trstmh/tru166 . 25359322

[46] Galatas B, Ly S, Duong V, Baisley K, Nguon K, Chan S, et al. Long-lasting immune protection and other epidemiological findings after chikungunya emergence in a Cambodian rural community, April 2012. PLoS Negl Trop Dis. 2016; 10(1): e0004281. doi: 10.1371/journal.pntd.0004281 . 26752630 PMC4713465

[47] de Souza WM, de Lima STS, Simões Mello LM, Candido DS, Buss L, Whittaker C, et al. Spatiotemporal dynamics and recurrence of chikungunya virus in Brazil: an epidemiological study. Lancet Microbe. 2023; 4(5): e319–e329. doi: 10.1016/S2666-5247(23)00033-2 . 37031687 PMC10281060

[48] Ammatawiyanon L, Tongkumchum P, McNeil D, Lim A. Statistical modeling for identifying chikungunya high-risk areas of two large-scale outbreaks in Thailand’s southernmost provinces. Sci Rep. 2023; 13(1): 18972. doi: 10.1038/s41598-023-45307-9 . 37923773 PMC10624817

[49] Bian G, Xu Y, Lu P, Xie Y, Xi Z. The endosymbiotic bacterium *Wolbachia* induces resistance to dengue virus in *Aedes aegypti*. PLoS Pathog. 2010; 6(4): e1000833. doi: 10.1371/journal.ppat.1000833 . 20368968 PMC2848556

[50] Hurk AFVD, Hall-Mendelin S, Pyke AT, Frentiu FD, McElroy KL, Day A, et al. Impact of *Wolbachia* on infection with chikungunya and yellow fever viruses in the mosquito vector *Aedes aegypti*. PLoS Negl Trop Dis. 2012; 6(11): e1892. doi: 10.1371/journal.pntd.0001892 . 23133693 PMC3486898

[51] Aliota MT, Walker EC, Uribe Yepes A, Dario Velez I, Christensen BM, Osorio JE. The *w*Mel strain of *Wolbachia* reduces transmission of chikungunya virus in *Aedes aegypti*. PLoS Negl Trop Dis. 2016; 10(4): e0004677. doi: 10.1371/journal.pntd.0004677 27124663 PMC4849757

[52] Pinto SB, Riback TIS, Sylvestre G, Costa G, Peixoto J, Dias FBS, et al. Effectiveness of *Wolbachia*-infected mosquito deployments in reducing the incidence of dengue and other *Aedes*-borne diseases in Niterói, Brazil: a quasi-experimental study. PLoS Negl Trop Dis. 2021; 15(7): e0009556. doi: 10.1371/journal.pntd.0009556 . 34252106 PMC8297942

[53] Raquin V, Valiente Moro C, Saucereau Y, Tran FH, Potier P, Mavingui P. Native *Wolbachia* from *Aedes albopictus* blocks chikungunya virus infection in cellulo. PLoS One. 2015; 10(4): e0125066. doi: 10.1371/journal.pone.0125066 . 25923352 PMC4414612

[54] Liang X, Tan CH, Sun Q, Zhang M, Wong PSJ, Li MI, Mak KW, et al. *Wolbachia w*AlbB remains stable in *Aedes aegypti* over 15 years but exhibits genetic background-dependent variation in virus blocking. PNAS Nexus. 2022; 1(4): pgac203. doi: 10.1093/pnasnexus/pgac203 . 36714832 PMC9802048

[55] Wang GH, Gamez S, Raban RR, Marshall JM, Alphey L, Li M, et al. Combating mosquito-borne diseases using genetic control technologies. Nat Commun. 2021; 12(1): 4388. doi: 10.1038/s41467-021-24654-z . 34282149 PMC8290041

[56] Md Iderus NH, Singh SSL, Ghazali SM, Zulkifli AA, Ghazali NAM, Lim MC, et al. The effects of the COVID-19 pandemic on dengue cases in Malaysia. Front Public Health. 2023; 11: 1213514. doi: 10.3389/fpubh.2023.1213514 . 37693699 PMC10484591

