# Supplementary figures and images for: A change in circulating chikungunya virus variant impacts Aedes aegypti vector competence and spatiotemporal distribution of disease in Malaysia
Source: PLoS Negl Trop Dis. 2024 Oct 31;18(10):e0012632. doi: 10.1371/journal.pntd.0012632 (PMC11556719; doi:10.1371/journal.pntd.0012632)

**S1 Figure.** Construction of infectious clones pCMV-p2020A (E1-226A) and pCMV-p2020V (E1-226V).

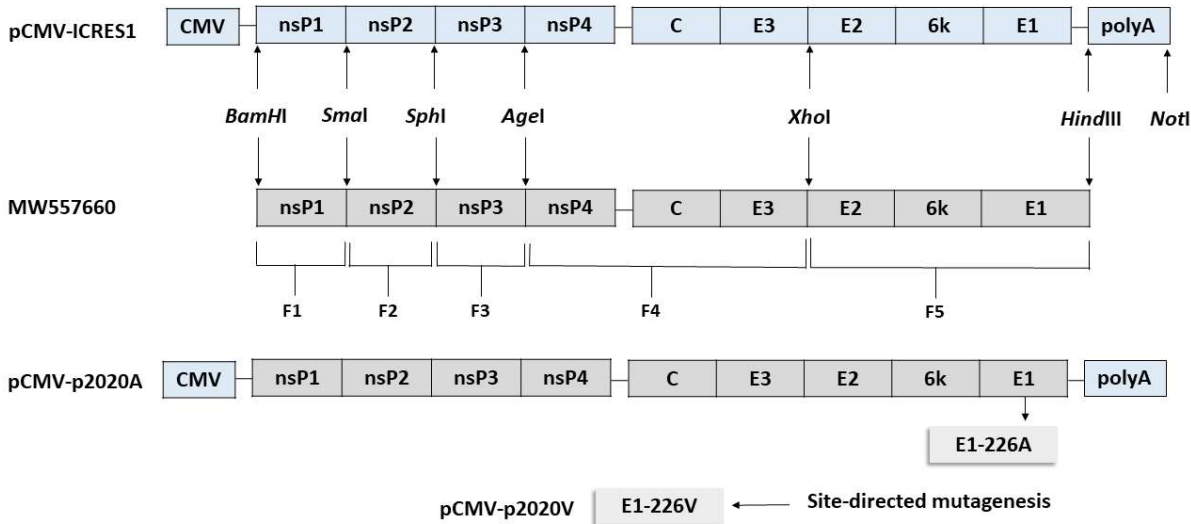

Supplement: S1 Fig — (PDF) [file pntd.0012632.s001.pdf]

S2 Figure. Incidence rates of notified CHIKV cases in Malaysia, 2009-2022.

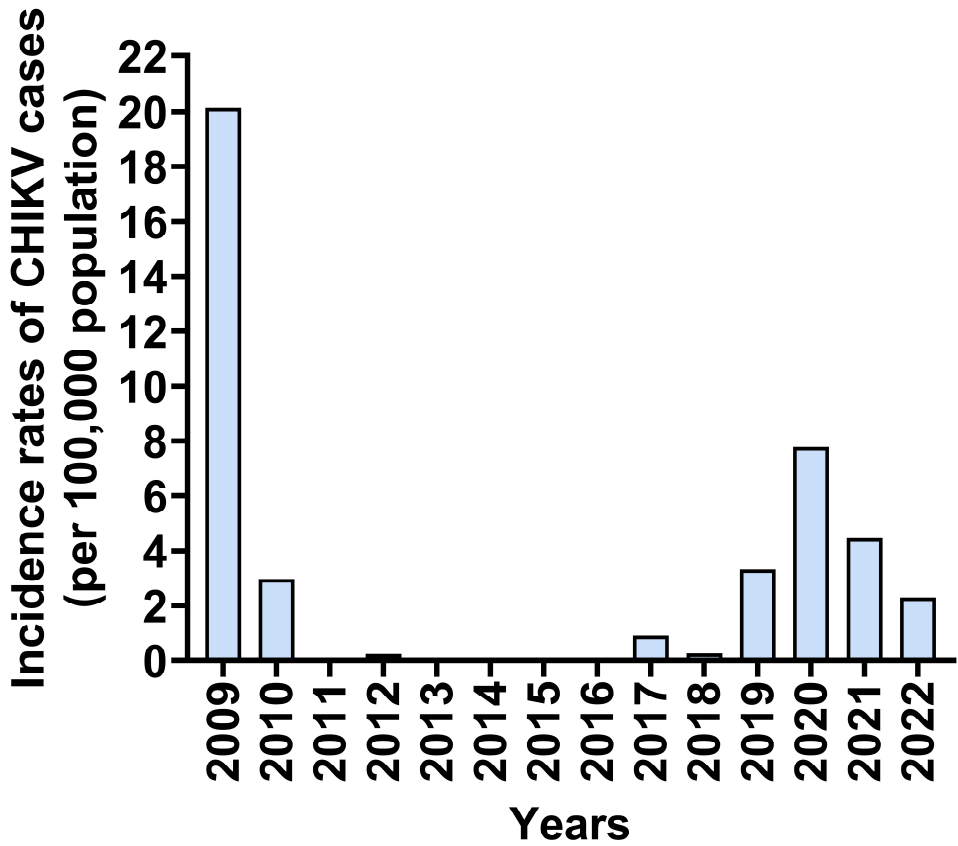

Supplement: S2 Fig — (PDF) [file pntd.0012632.s002.pdf]
