# Supplementary material for: A change in circulating chikungunya virus variant impacts Aedes aegypti vector competence and spatiotemporal distribution of disease in Malaysia
Source: PLoS Negl Trop Dis. 2024 Oct 31;18(10):e0012632. doi: 10.1371/journal.pntd.0012632 (PMC11556719; doi:10.1371/journal.pntd.0012632)

S3 Figure. Spatiotemporal dynamics of notified CHIKV cases in Malaysia between 2009-2022.

Cases are categorised as rural (green circles) or urban (red circles) based on Department of Statistics Malaysia criteria. Annual notified cases are shown in brackets. Rural cases were observed predominantly in 2009 to 2010. However, starting from 2017, there has been a noticeable shift of cases towards urban areas. The basemap of Malaysia was obtained from the Database of Global Administrative Areas (https://gadm.org/download_country36.html) and is freely available for academic use.

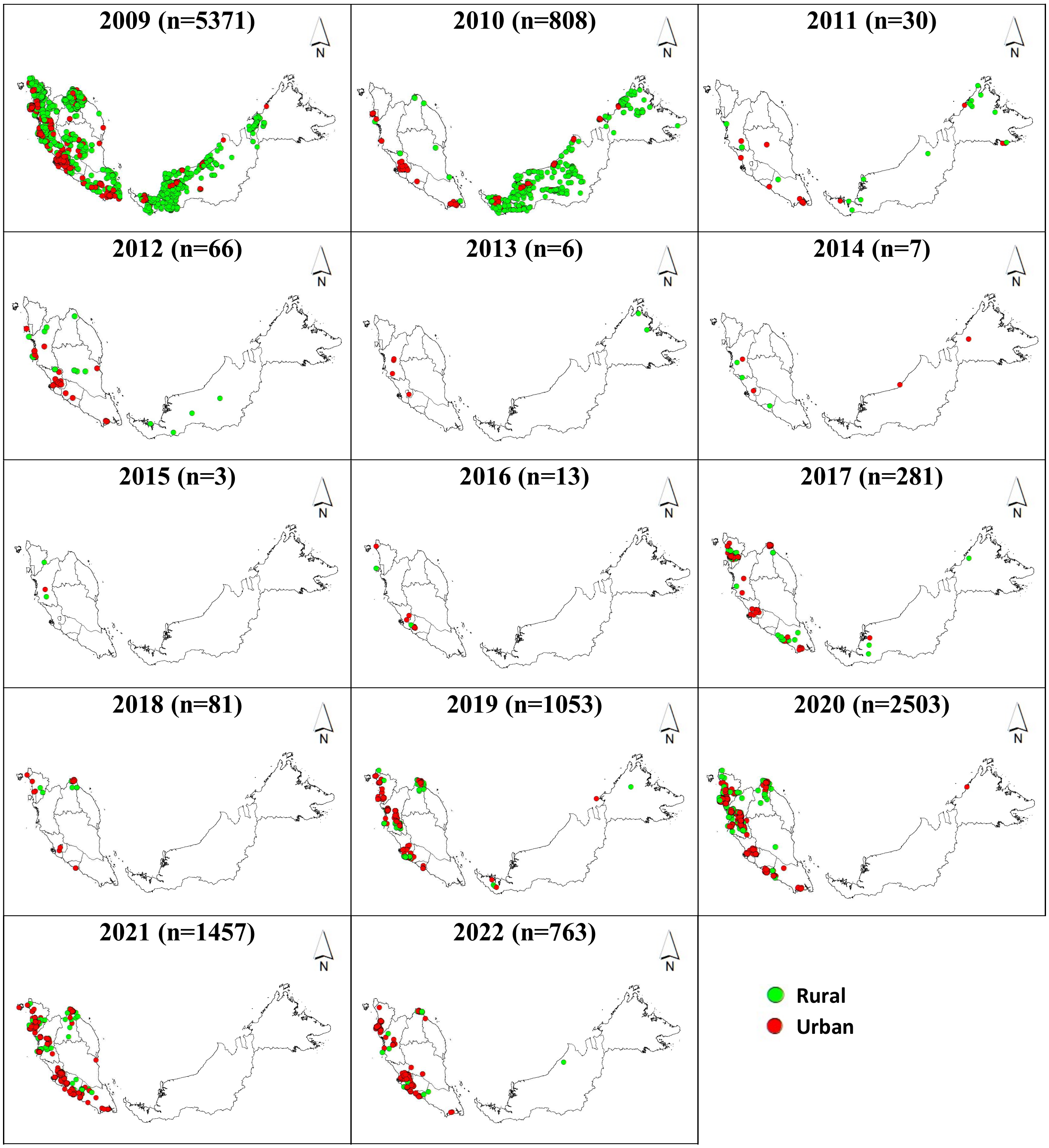

Supplement: S3 Fig — (PDF) [file pntd.0012632.s003.pdf]
