# Supplementary material for: A change in circulating chikungunya virus variant impacts Aedes aegypti vector competence and spatiotemporal distribution of disease in Malaysia
Source: PLoS Negl Trop Dis. 2024 Oct 31;18(10):e0012632. doi: 10.1371/journal.pntd.0012632 (PMC11556719; doi:10.1371/journal.pntd.0012632)
Supplement: S1 Table — (PDF) [file pntd.0012632.s004.pdf]

S1 Table. Sequences from recently reported CHIKV cases in Malaysia.

Strain name	Date of specimen	Place of origin	Host	Genotype	GenBank accession number
MY/2017/0040	June 2017	Kuala Lumpur, Malaysia	Human	ECSA	MW588418
MY/2017/2968	June 2017	Kuala Lumpur, Malaysia	Human	ECSA	MW588419
MY/2017/M/CHIK/17/07/02	July 2017	Kota Bahru, Kelantan, Malaysia	Human	ECSA	OR412349
MY/2017/M/CHIK/17/08/07	August 2017	Kota Bahru, Kelantan, Malaysia	Human	ECSA	OR412350
MY/2020/3092151	September 2020	Kuala Lumpur, Malaysia	Human	ECSA	MW557660
MY/2020/3092435	September 2020	Kuala Lumpur, Malaysia	Human	ECSA	MW557661
MY/2021/6988-3216386	December 2021	Kuala Lumpur, Malaysia	Human	ECSA	OR412347
MY/2021/7236-2509922	November 2021	Kuala Lumpur, Malaysia	Human	ECSA	OR412348
