# Supplementary material for: A change in circulating chikungunya virus variant impacts Aedes aegypti vector competence and spatiotemporal distribution of disease in Malaysia
Source: PLoS Negl Trop Dis. 2024 Oct 31;18(10):e0012632. doi: 10.1371/journal.pntd.0012632 (PMC11556719; doi:10.1371/journal.pntd.0012632)
Supplement: S2 Table — (PDF) [file pntd.0012632.s005.pdf]

S2 Table. PCR primers for amplification of five CHIKV fragments for cloning.

The reaction mixture contained 0.5 µM forward primer, 0.5 µM reverse primer, 200 µM dNTP (Thermo Scientific, USA), 0.01 U Q5 High-Fidelity DNA Polymerase (New England Biolabs, USA), 1X Q5 Reaction Buffer (NEB, USA), 1 ng of cDNA, and nuclease-free water to a final volume of 50 µl. PCR cycling conditions were: initial denaturation at 98°C for 30 secs; 40 cycles of denaturation at 98°C for 10 secs, annealing at 64°C for 30 secs, and extension at 72°C for 30 secs/kb; followed by final extension at 72°C for 5 mins.

**Fragment**	**Targeted sites (nucleotides)**	**Forward primer (F)**	**Reverse primer (R)**	**Expected amplicon sizes (base pairs)**
Fragment 1	18 - 2227	5'-CACGTAGCCTACCAGTTTCTTA-3'	5'- ATGACTGCAATTTTGTATGGGC-3'	2200
Fragment 2	1412 - 3542	5'- CGAGTTTGACAGCTTTGTGGTA-3'	5'- CTTTTACTGGGCGGTGTTCG-3'	2130
Fragment 3	2577 - 5874	5'- AACATCTGCACCCAAGTGTACC-3'	5'- TCTACTTTGCGCGACTGATACC-3'	3297
Fragment 4	5630 - 10130	5'- ACGGACGACGAGTTACGACTAG-3'	5'- AAAGGTTGCTGCTCGTTCCAC-3'	4500
Fragment 5	9093 - 11793	5'- AGTCCGGCAACGTAAAGATCAC-3'	5'- TACGTCCCTGTGGGTTCG-3'	2700
